# Complexities in the Diagnostic Evaluation and Treatment of Infant Hemophagocytic Lymphohistiocytosis

**DOI:** 10.3390/hematolrep18040051

**Published:** 2026-07-24

**Authors:** Reeja Raj, Ramya Ramakrishnan, Carlos Vargas, Muhammad Khalid, Gopi Mohan, David McCall, Amber Gibson, Branko Cuglievan, Cesar Nunez, Brandon Brown, Monica Bray, Miriam B. Garcia

**Affiliations:** 1 Department of Pediatrics, The University of Texas MD Anderson Cancer Center, 1515 Holcombe Blvd., Unit 1487, Houston, TX 77030, USA; gmohan@mdanderson.org (G.M.);; 2 Department of Pediatrics, Oklahoma University Health Sciences Center, 1100 N Lindsay Ave., Oklahoma City, OK 73104, USA; 3 Department of Pediatrics, Texas Children’s Hospital, 6701 Fannin St., Houston, TX 77030, USA; 4 Department of Pathology and Laboratory Medicine, McGovern Medical School, UTHealth, 64313 Fannin St., Houston, TX 77030, USA; 5 Department of Pediatrics, McGovern Medical School, UTHealth, 64313 Fannin St., Houston, TX 77030, USA

**Keywords:** pediatrics, hemophagocytic lymphohistiocytosis, genetics, immunomodulation, immune dysregulation

## Abstract

Background and Clinical Significance: Hemophagocytic lymphohistiocytosis (HLH) is a life-threatening hyperinflammatory syndrome characterized by excessive activation of the immune system. Case Presentation: We present the case of an eight-month-old infant with influenza A(H1N1) who presented with seizures, hepatosplenomegaly, cytopenias, and markedly elevated levels of ferritin, IL-18, and CXCL9, raising concern for HLH. Despite initiation of HLH-directed and antiviral therapy, she succumbed to cardiorespiratory failure. Genetic testing revealed heterozygous variants in *LYST* and *NLRC4,* suggesting a potential genetic predisposition. Conclusions: This case underscores the challenge of distinguishing primary from secondary HLH and highlights the importance of balancing aggressive immunosuppression with infection control. Early genetic and cytokine profiling may assist in guiding personalized treatment strategies, enabling targeted interventions that avoid excessive immunosuppressive treatment in cases of reactive hyperinflammation while facilitating timely escalation of therapy in true or refractory HLH.

## 1. Introduction

Hemophagocytic lymphohistiocytosis (HLH) is a rare, life-threatening hyperinflammatory syndrome caused by immune dysregulation, often progressing to organ failure and death if untreated. It occurs across all age groups and is difficult to diagnose due to its nonspecific features. HLH is classified as primary or secondary [1,2]. Primary HLH arises from germline mutations impairing cytotoxic T-cell and natural killer (NK) cell function [3,4,5]. Impaired clearance of antigenic stimuli drives persistent macrophage activation and cytokine overproduction [6]. Secondary, or acquired HLH, has a similar inflammatory cascade but no known monogenic cause. It may be triggered by infection [7,8], autoimmune diseases such as systemic juvenile idiopathic arthritis (sJIA) [9,10,11], or malignancies, particularly hematologic malignancies [12,13].

Diagnosis is guided by the HLH-2004 criteria, but their overlap with severe infections or sepsis often leads to delays in identification [14,15]. The H-score is another clinical scoring system validated in adults and designed to estimate the likelihood of reactive HLH, especially in the setting of sepsis or infection. It is less commonly used in pediatrics, though it may be adapted for early assessment or screening [16] (Supplemental Table S1).

The standard treatment for HLH remains the HLH-2004 treatment, which includes dexamethasone and etoposide, with hematopoietic stem cell transplantation for refractory or genetic disease. Emerging therapies include anakinra (a recombinant IL-1 receptor antagonist) and ruxolitinib (a JAK inhibitor), while emapalumab (an anti-IFN-γ monoclonal antibody) is FDA-approved for primary HLH [4,17].

Not all patients who meet the HLH-2004 criteria require HLH-2004 treatment. The HLH-2004 diagnostic criteria lack formal validation, and many severe inflammatory conditions can meet these diagnostic thresholds without representing true HLH [18]. Conversely, some patients may not fulfill the diagnostic criteria until they are critically ill, highlighting the need for a high index of suspicion. Clinicians must distinguish early on which patients are likely to respond to less intensive immunomodulation from those most likely to benefit from initiation of HLH-2004 [1].

## 2. Case

An eight-month-old previously healthy full-term female infant presented to the pediatric critical care unit with febrile seizures in the setting of influenza A(H1N1) infection. She did not initially have severe respiratory distress on presentation and was electively intubated due to persistent seizures.

On physical examination, the patient was found to have significant pallor, hepatosplenomegaly, and palpable lymphadenopathy. Initial labs showed a hemoglobin level of 9.7 g/dL, platelets of 206 × 10^9^/L, and a white blood cell count of 39.3 × 10^9^/L with 13% bands. Laboratory testing revealed fasting triglycerides of 330 mg/dL, fibrinogen of 77 mg/dL, soluble IL-2 receptor (sIL-2R) of 16,542 pg/mL, and low NK cell subsets. Send-out testing revealed a low perforin-positive NK cell assay and normal granzyme B expression studies. CD107a mobilization testing could not be completed due to insufficient sample quantity. Additional labs obtained also showed elevated inflammatory markers: ferritin of 19,000 ng/mL (normal 10–100 ng/mL) [19], IL-18 of 250,000 pg/mL (normal < 300 pg/mL) [20], and CXCL9 of 48,091 pg/mL (normal < 39.0 pg/mL) [20,21]. Initial cytokine testing showed an elevation in IFN-γ to 5 pg/mL, above the institutional reference range of <1 pg/mL. A respiratory viral panel was positive for influenza A(H1N1). Medical, family, and psychosocial histories, including relevant genetic information, were noncontributory; no family history of malignancy or rheumatologic disease was reported.

Imaging studies confirmed hepatosplenomegaly with bilateral axillary, para-aortic, iliac, and inguinal lymphadenopathy and no other masses on computed tomography (CT) of the abdomen and pelvis. Chest X-ray (CXR) revealed patchy multifocal airspace opacities consistent with atelectasis and/or edema as well as a trace right pleural effusion. Magnetic resonance imaging (MRI) of the brain showed no leptomeningeal enhancement of acute intraparenchymal lesions but did show pachymeningeal enhancement in the bilateral tectorial leaflets. Bone marrow aspirate and biopsy, lymph node biopsy, and cerebrospinal fluid (CSF) cytology were all negative for malignancy, though the CSF neopterin level was elevated. No hemophagocytosis was noted on bone marrow studies. Her H-score was 238 points, which translated into a 98–99% probability of HLH, and she met six of the eight HLH-2004 diagnostic criteria (Supplemental Table S1), except for hemophagocytosis. Given her infantile age, the differential included, alongside influenza, HLH (primary or secondary) and autoinflammatory disorders such as systemic juvenile idiopathic arthritis (sJIA) or an inflammasome disease, given the markedly elevated IL-18 levels.

Genetic testing showed that she had a positive heterozygous missense variant (c.8913T>G, p.Asn2971Lys) in exon 35 of the *LYST* gene and a heterozygous missense variant (c.478G>A, p.Ala160Thr) in exon 4 of the *NLRC4* gene, a heterozygous synonymous variant (c.876C>T, p.Ser292Ser) in exon 10 of the AP3D1 gene and a heterozygous intronic variant (chr1:22081719, c.106-3T>C) 3 bp before the start of exon 3 in the CDC42 gene. All variants were classified as variants of unknown significance (VUS) according to the American College of Medical Genetics and Genomics criteria.

**Figure 1:**
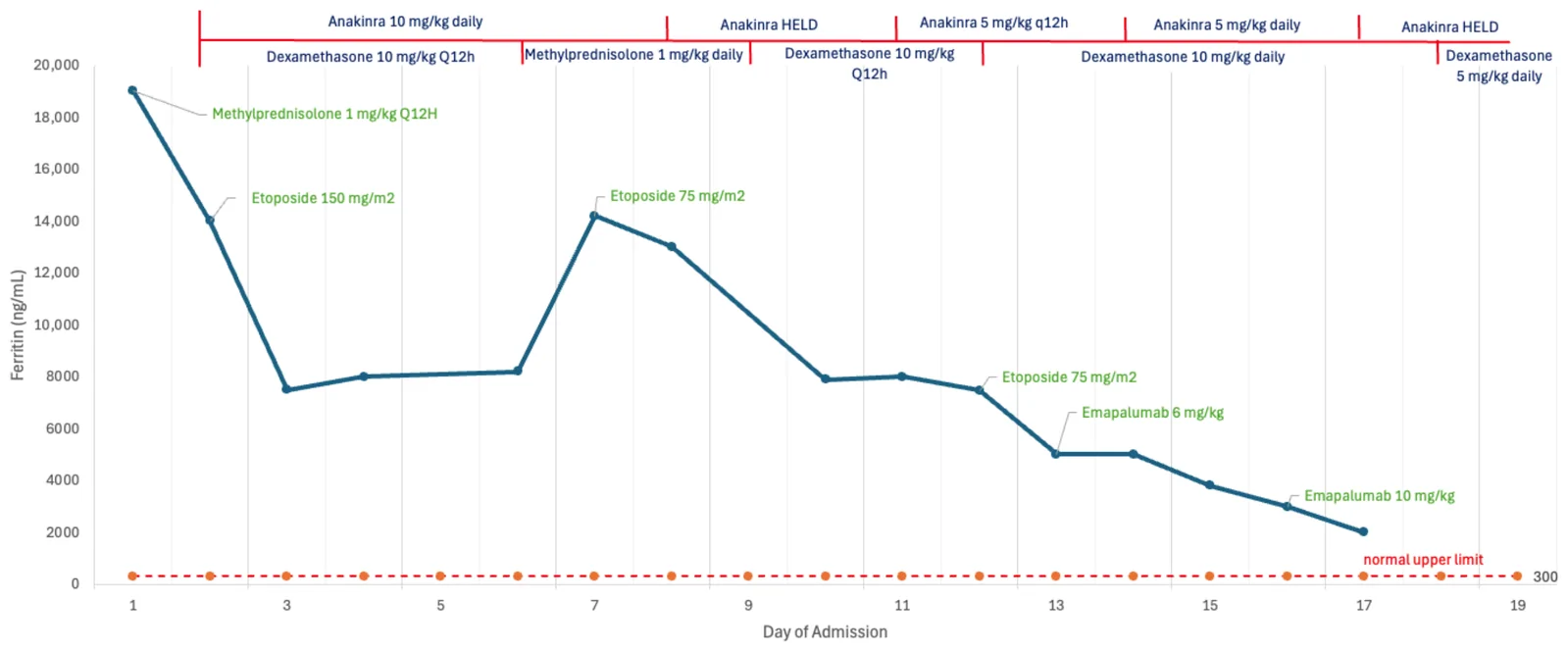
Ferritin trend over time with treatment interventions. This figure depicts ferritin levels measured at serial time points during treatment, highlighting changes over the course of therapy and serving as a marker of disease activity and treatment response.

She was started on dexamethasone and etoposide as per the HLH-2004 protocol. Anakinra was also started at diagnosis. Etoposide was later found to decrease the risk of further immunosuppression in the setting of infection. Total IFN-γ levels, however, continued to rise, and, due to concern for underlying primary HLH, emapalumab-Izsg was initiated approximately a week later (Figure 1). Intrathecal methotrexate was administered due to neopterin positivity in CSF. Following initiation of HLH-directed therapy, repeat inflammatory testing approximately two weeks later showed a CXCL9 level of 1464 pg/mL. The patient tested positive for H1N1 at admission and again on days 13 and 19. She completed a 10-day course of oseltamivir and continued receiving it prophylactically due to suspected immunodeficiency. Ruxolitinib was planned but not administered before death.

**Figure 2:**
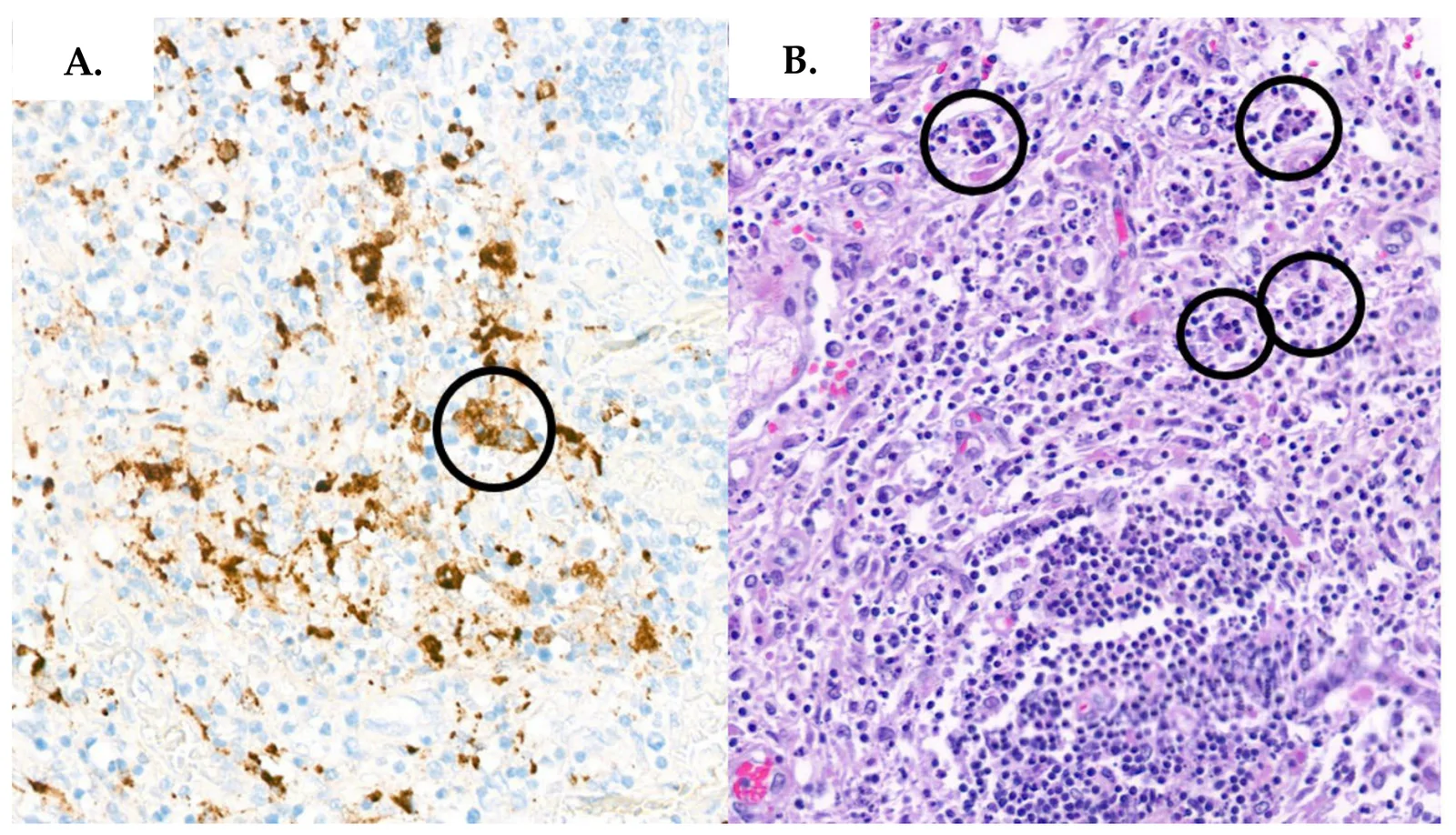
Hemophagocytes from lung hilar lymph node. (**A**) CD68 immunohistochemical staining demonstrates macrophages with cytoplasmic and membranous brown staining; a representative hemophagocyte is circled. (**B**) Hematoxylin and eosin-stained section of the hilar lymph node showing representative hemophagocytes, circled. Both panels were imaged at 20× magnification.

Despite improvement in inflammatory markers, the patient died from progressive cardiorespiratory failure. A limited autopsy revealed severe viral lung pathology with histologic findings of hemophagocytes consistent with underlying HLH (Figure 2).

## 3. Discussion

This case illustrates the complexity of managing a critically ill infant with HLH features, in whom balancing infection control and immunosuppression is challenging. The classic distinction between primary and secondary HLH, based on the absence or presence of known genetic mutations, is increasingly viewed as a spectrum of hyperinflammatory states shaped by both genetic predisposition and environmental triggers [1]. Some patients carry biallelic mutations that almost “fatalistically” predispose them to HLH, while others may harbor milder or heterozygous variants that still confer increased vulnerability during significant inflammatory events.

Although this patient lacked pathogenic mutations (e.g., *PRF1, UNC13D*) found in classic HLH genes, testing identified variants in *LYST* and *NLRC4,* which may carry a high risk of affecting protein function, as both are associated with macrophage hyperactivation [22,23]. However, both variants were heterozygous and classified as VUS and should therefore be interpreted cautiously. *LYST* encodes a lysosomal trafficking regulator that is important for cytotoxic T-cell and NK-cell granule function. *LYST*-associated disease is typically inherited in an autosomal recessive manner; therefore, the clinical relevance of this single heterozygous missense VUS is uncertain. Although the LYST gene mutation may be associated with impaired CD107a mobilization, CD107a could not be assessed in this patient; however, NK cell subsets were lower than normal. Regardless, CD107a expression or abnormal NK cell numbers alone would not fully explain the observed hyperinflammatory phenotype. A previous report described a *LYST* mutation in severe influenza A(H1N1) infection, providing biologic context but not establishing a causal role for the variant identified here [24,25]. *NLRC4* variants have been associated with severe outcomes in influenza A(H1N1) infection through inflammasome activation and IL-18 overproduction [25]. However, it is uncertain whether the specific *NLRC4* p.Ala160Thr variant localizes to a domain that is associated with known gain-of-function MAS variants. Published data from Steiner et al. suggest that the p.Ala160Thr variant may have a more limited, stimulus-dependent functional effect than previously established dominant gain-of-function *NLRC4* variants and has been associated with recessive autoinflammation or, in heterozygous carriers, increased susceptibility to ulcerative colitis rather than with a definitive MAS phenotype [26]. Thus, although the marked IL-18 elevation in this patient raises the possibility of inflammasome dysregulation, the contribution of this heterozygous *NLRC4* variant to the clinical course remains speculative.

Markedly elevated IL-18 and CXCL9 levels supported a hyperinflammatory process beyond an uncomplicated influenza infection. IL-18 has been linked to inflammasome activation and sJIA-associated macrophage activation syndrome (MAS), while CXCL9 reflects IFN-γ-driven immune activation, a central pathway in MAS, and secondary HLH. In this context, influenza was suspected to have served as an infectious trigger that unmasked an underlying autoinflammatory susceptibility, such as inflammasome disorder or occult sJIA. Recognizing this pattern is important because MAS can deteriorate rapidly and often requires prompt identification and treatment in addition to antiviral and supportive care [27,28,29,30].

IL-18 is upstream of IFN-γ, which directly induces CXCL9, a pharmacodynamic surrogate marker of IFN-γ pathway activation, and drives hemophagocytosis and cytopenias [31]. Interpretation of serum IFN-γ levels after treatment can be challenging because available assays may not reliably distinguish free circulating IFN-γ from antibody-bound IFN-γ following IFN-γ-directed therapy. Therefore, CXCL9 may provide a more informative marker of ongoing IFN-γ signaling. Emapalumab binds and neutralizes IFN-γ, preventing receptor activation and subsequent downstream signaling. If IFN-γ blockade is effective, CXCL9 production would be expected to decrease over time, reflecting reduced pathway activation. Given this mechanism, emapalumab was initiated prior to receipt of genetic results to directly bind and neutralize circulating IFN-γ. By shutting down the IFN-γ-driven inflammatory cascade, downstream JAK-STAT signaling can be effectively blocked to prevent further macrophage activation and related inflammatory organ damage [32,33]. While only FDA-approved for primary HLH, emapalumab is mechanistically appropriate in both primary and secondary HLH and was utilized for this patient, who exhibited features of both, and it should be considered in similar patients with inflammation-related organ involvement and secondary HLH. In this case, despite targeted therapy, the patient’s course was complicated by viral persistence and respiratory failure that ultimately led to her deterioration.

Although standard HLH-directed therapy (with etoposide and high-dose corticosteroids) can be lifesaving when immune hyperactivation is the primary driver of organ dysfunction [17], systemic immunosuppression may also be detrimental in the setting of a concomitant active infection, as observed in our patient. This concern is particularly relevant in influenza, for which adjunctive corticosteroid therapy has been associated in observational studies and meta-analyses with higher mortality, increased nosocomial infections, and delayed viral clearance. Accordingly, the Infectious Diseases Society of America (IDSA) guidelines advise against routine corticosteroid use for influenza infection [34,35]. Etoposide may confer additional risk through myelosuppression, including neutropenia and lymphocyte depletion, potentially impairing control of the primary viral infection and increasing susceptibility to secondary bacterial or fungal infections. In this case, these considerations support a more measured approach focused on treatment of the infectious trigger and the use of targeted immunomodulation when feasible, rather than the immediate initiation of broad cytotoxic HLH-directed therapy.

For this patient, CSF cytology was negative, and brain MRI did not demonstrate leptomeningeal enhancement; however, the markedly elevated CSF neopterin level raised concern for possible CNS immune activation in the setting of severe systemic HLH. CSF neopterin reflects intrathecal macrophage activation and has been reported to be substantially elevated in children with familial HLH and neurologic involvement; in an early series, higher levels were associated with active CNS disease and remained elevated in a patient with progressive fatal illness, whereas levels declined with clinical remission [36]. More recently, pediatric cases of CNS-restricted familial HLH similarly demonstrated elevated CSF neopterin at presentation, with normalization after disease control, supporting its potential role as a marker of CNS inflammatory activity rather than a stand-alone diagnostic test [37]. Given the patient’s rapidly progressive hyperinflammatory syndrome and concern that early or occult CNS-HLH may not be fully excluded by negative cytology or the absence of leptomeningeal enhancement, intrathecal methotrexate was administered as part of the HLH-directed treatment strategy. This decision was based on concern for possible CNS involvement rather than on the CSF neopterin level alone.

HLH may also represent a clinical manifestation of inborn errors of immunity (IEI) beyond the classical familial HLH genes. While defects in cytotoxic lymphocyte function remain central to familial HLH, emerging literature highlights that HLH can occur in patients with IEI involving immune dysregulation, impaired pathogen control, autoinflammation, and abnormal cytokine activation [38]. Similarly, a recent systematic review of pediatric HLH occurring in IEI other than familial HLH emphasized that HLH may serve as an important warning sign for an underlying immune disorder, particularly in children with severe, recurrent, infection-triggered, or treatment-refractory hyperinflammation [39]. Our patient’s presentation with severe influenza-associated HLH and VUSs in immune-dysregulation genes underscores the diagnostic challenges of distinguishing infection-triggered HLH from an underlying genetic or autoinflammatory predisposition. This broader framework supports consideration of expanded immune and genetic evaluation in select pediatric HLH cases, while recognizing that the pathogenic relevance of individual variants requires functional confirmation.

Several limitations should be considered when interpreting the findings of this case. This report describes a single patient, and as such, its findings may not be generalizable to other patients with influenza-associated HLH. Another limitation is the lack of comprehensive functional studies to clarify the potential pathogenic and clinical significance of the identified heterozygous *LYST* and *NLRC4* variants, which are currently classified as VUSs. Accordingly, definitive conclusions regarding their causal role or contribution to genetic predisposition cannot be drawn. Specifically, inflammasome-focused assays, such as measurements of IL-18/IL-1β secretion, caspase-1 activation, or apoptosis-associated speck-like protein containing a CARD (ASC) speck formation following stimulation, were not performed to support the pathogenicity of the *NLRC4* variant. Population frequencies and additional in silico pathogenicity estimates were not available for inclusion, and parental testing could not be performed. Therefore, the inheritance pattern and phase of the identified variants could not be established. As such, these variants remain classified as VUSs, and their contribution to the patient’s hyperinflammatory clinical picture cannot be definitively established.

Although this patient received cytokine-directed therapies, including anakinra and emapalumab, treatment was not guided by a confirmed molecular diagnosis, and no novel genotype-specific therapy was administered. Consequently, discussion of additional targeted therapeutic strategies remains theoretical and informed by previously published experience. This clinical course nevertheless highlights the challenges of selecting immunomodulatory therapy when severe hyperinflammation coexists with persistent viral infection and an uncertain genetic background. Furthermore, although elevated IL-18 in *NLRC4*-associated inflammation, *LYST* variants in virus-triggered HLH, and cytokine profiling in hyperinflammatory disorders have each been previously described, this case underscores the difficulty of integrating these findings when genetic variants of uncertain significance and treatment decisions must be made during rapidly progressive critical illness.

Many proinflammatory conditions can meet HLH diagnostic criteria without necessarily requiring treatment with immunosuppressive chemotherapy as per HLH-2004 [25]. The diagnostic criteria created for the HLH-2004 clinical trial should not be heavily relied on due to their lack of specificity. Even when the criteria are met, or HLH is diagnosed, identifying underlying triggers, whether they are underlying malignancies, infections, and/or autoimmune diseases, is important for overall management [40].

This case highlights several clinically relevant lessons for the recognition and management of suspected HLH in infants and children. In the setting of severe infection, the combination of persistent fever, worsening cytopenias, hepatic dysfunction, coagulopathy, markedly elevated ferritin, and evolving organ failure should raise concern for a hyperinflammatory syndrome and prompt early coordination among pediatric hematology-oncology, immunology, infectious diseases, and critical care specialists. Diagnostic studies should be pursued concurrently with treatment, including assessment for infectious precipitants, trending of inflammatory and end-organ injury markers, and consideration of immunologic and genetic testing, particularly for infants and for patients with severe, recurrent, atypical, or treatment-resistant inflammation. Although no causal relationship can be assigned to the identified genetic variants, the case reinforces the need to consider underlying immune dysregulation when hyperinflammation is unusually severe or disproportionate to the infectious trigger. Treatment should be tailored to the clinical trajectory and suspected inflammatory mechanism, while maintaining active management of the underlying infection and avoiding delays in anti-inflammatory therapy when HLH is strongly suspected.

## 4. Conclusions

Anchoring on HLH-2004 may divert attention from organ-directed therapy, cause excessive immunosuppression, impair infection control, and lead to significant complications. This patient’s clinical course underscores the importance of integrating genetic analysis, cytokine profiling, and attentive infection monitoring when managing HLH. Heightened vigilance in patients with HLH is critical, as immunosuppression can reveal or exacerbate infections, necessitating careful monitoring, targeted immunomodulatory and antimicrobial therapy, and meticulous management of end-organ damage.

## Data Availability

The original contributions presented in this study are included in the article. Further inquiries can be directed to the corresponding authors.

## Supplementary Materials

The following supporting information can be downloaded at: https://www.mdpi.com/article/10.3390/hematolrep18040051/s1, Table S1: Diagnostic Criteria and HScore Parameters for Hemophagocytic Lymphohistiocytosis.

## Abbreviations

The following abbreviations are used in this manuscript:

HLH	Hemophagocytic lymphohistiocytosis
sIL-2R	soluble IL-2 Receptor
NK	Natural Killer
CT	Computed tomography
CXR	Chest X-ray
MRI	magnetic resonance imaging
sJIA	systemic juvenile idiopathic arthritis
CSF	cerebral spinal fluid
VUS	variants of unknown significance
MAS	macrophage activation syndrome
IDSA	Infectious Diseases Society of America
