# Supplementary material for: Complexities in the Diagnostic Evaluation and Treatment of Infant Hemophagocytic Lymphohistiocytosis
Source: Hematol Rep. 2026 Jul 24;18(4):51. doi: 10.3390/hematolrep18040051 (PMC13511909; doi:10.3390/hematolrep18040051)
Supplement: Supplementary file 1 [file hematolrep-18-00051-s001.zip › hematolrep-4287910-supplementary.pdf]

Supplemental Table S1: Diagnostic Criteria and HScore Parameters for Hemophagocytic Lymphohistiocytosis

Shown are the standard diagnostic criteria for HLH alongside the parameters included in the HScore, which may aid in assessing the probability of HLH in the appropriate clinical setting [15,16]. Criteria met by the patient are indicated.

**HLH 2004 Criterion**	**Definition**	**Patient Finding**	**Criteria Met**
Fever	Temperature ≥38.5°C, persistent	Persistent fever at presentation in the setting of influenza infection	Yes
Splenomegaly	Clinically detectable enlargement of the spleen	Hepatosplenomegaly on physical examination and imaging	Yes
Cytopenias	Hemoglobin <9 g/dL; Platelets <100,000/µL; Neutrophils <1,000/µL	Hemoglobin 9.7 g/dL, platelets 206 x10^9/L, and white blood cells 39.3 x10^9/L with 13% bands	No
Hypertriglyceridemia and/or Hypofibrinogenemia	Fasting triglycerides ≥265 mg/dL and/or fibrinogen ≤150 mg/dL	Fasting triglycerides: 330 mg/dL; fibrinogen: 77 mg/dL	Yes
Hemophagocytosis	Evidence in bone marrow, spleen, lymph nodes, or liver (no evidence of malignancy required)	Not identified	No
Low or Absent NK Cell Activity	Decreased or absent natural killer (NK) cell cytotoxic function	Low NK cell subsets; CD107a mobilization sample insufficient for testing	Yes
Elevated Ferritin	Ferritin ≥500 ng/mL (often significantly higher in HLH)	Ferritin: 19,000 ng/mL	Yes
Elevated Soluble IL-2 Receptor (sCD25)	Soluble CD25 (sIL-2R) ≥2 standard deviations above age-adjusted normal values	Soluble IL-2R: 16,542 pg/mL	Yes
**HScore**	Composite weighted score for reactive/secondary HLH based on 9 parameters: known	Hscore: 238	Yes

	underlying immunosuppression, temperature, organomegaly, number of cytopenias, ferritin, triglycerides, fibrinogen, AST/SGOT, and hemophagocytosis on bone marrow aspirate. Higher scores indicate a greater probability of HLH; scores ≥169 support the diagnosis.		
**Other**			
Genes associated with primary HLH	Biallelic pathogenic or likely pathogenic variants in PRF1, UNC13D, STX11, or STXBP2<br><br>OR hemizygous pathogenic variant in X-linked forms, including SH2D1A or XIAP	Not identified.	No
Functional immunologic assays	Functionally validated defect	Low perforin positive NK cells.<br><br>Normal granzyme B.<br><br>Not performed - IL-1β secretion, caspase-1 activation, or apoptosis-associated speck-like protein containing a CARD (ASC) speck formation following stimulation.	No

Note: Diagnosis of HLH requires either a molecular diagnosis consistent with HLH or fulfillment of five out of the eight diagnostic criteria listed. The HScore is not part of the HLH-2004 criteria but may be used as an adjunctive tool to estimate the probability of reactive/secondary HLH.
